# Cross-border mobility in European countries*:* associations between cross-border worker status and health outcomes

**DOI:** 10.1186/s12889-021-10564-8

**Published:** 2021-03-24

**Authors:** Lucas Nonnenmacher, Michèle Baumann, Etienne le Bihan, Philippe Askenazy, Louis Chauvel

**Affiliations:** 1 Institute for Research on Sociology and Economic Inequalities. Department of Social Sciences, University of Luxembourg, Belval Campus, L-4366 Esch-sur-Alzette, Luxembourg; 2 Centre Maurice Halbwachs, CNRS-ENS-PSL, 48 Boulevard Jourdan, 75014 Paris, France

**Keywords:** Cross-border workers, Physical health, Self-perceived health, Health index

## Abstract

**Background:**

Mobility of workers living in one country and working in a different country has increased in the European Union. Exposed to commuting factors, cross-border workers (CBWs) constitute a potential high-risk population. But the relationships between health and commuting abroad are under-documented. Our aims were to: (1) measure the prevalence of the perceived health status and the physical health outcomes (activity limitation, chronic diseases, disability and no leisure activities), (2) analyse their associations with commuting status as well as (3) with income and health index among CBWs.

**Methods:**

Based on the ‘*Enquête Emploi*’, the French cross-sectional survey segment of the European Labour Force Survey (EU LFS), the population was composed of 2,546,802 workers. Inclusion criteria for the samples were aged between 20 and 60 years and living in the French cross-border departments of Germany, Belgium, Switzerland and Luxembourg. The Health Index is an additional measure obtained with five health variables. A logistic model was used to estimate the odds ratios of each group of CBWs, taking non-cross border workers (NCBWs) as the reference group, controlling by demographic background and labour status variables.

**Results:**

A sample of 22,828 observations (2456 CBWs vs. 20,372 NCBWs) was retained. The CBW status is negatively associated with chronic diseases and disability. A marginal improvement of the health index is correlated with a wage premium for both NCBWs and CBWs. Commuters to Luxembourg have the best health outcomes, whereas commuters to Germany the worst.

**Conclusion:**

CBWs are healthier and have more income. Interpretations suggest (1) a healthy cross-border phenomenon steming from a social selection and a positive association between income and the health index is confirmed; (2) the existence of major health disparities among CBWs; and (3) the rejection of the spillover phenomenon assumption for CBWs. The newly founded European Labour Authority (ELA) should take into account health policies as a promising way to support the cross-border mobility within the European Union.

**Supplementary Information:**

The online version contains supplementary material available at 10.1186/s12889-021-10564-8.

## Background

Between 2017 and 2018, the number of cross-border workers (CBWs) increased from 1.4 million to 1.5 million workers in the European Union (EU) [1], making cross-border mobility a phenomenon of growing interest. Since freedom of movement for workers has been defined as the cornerstone of the EU, commuting abroad became a preoccupation of and major challenge for the states such as transport (Bike2Work EU’s project of 2014–2017) (ESPN 2019) but also for the European Public Health and Social Policy a major challenge. Commuters have less time to participate in activities that are beneficial for their health such as sport, meditation or socialising [2]. The link between CBWs and health was mainly addressed regarding the commuters’ access to the healthcare system [3].

CBWs are experiencing a specific and common lifestyle, while commuting more than the rest of the workforce [4]. Commuting may affect workers’ health, and projections indicate that more and more workers will be commuting abroad in the future [5]. Therefore, CBWs represent a potential high-risk population combining several drawbacks which may impact their health. European Social Policy might be soon confronted with a declining health status of workers leading to a rise of health expenditures. In this perspective, an increased understanding of cross-border health issues is a matter of prime importance. Consequently, our main research question is: do health disparities between Cross-Border Workers (CBWs) and Non-Cross-Border Workers (NCWBs) exist?

Scientific works have tried to capture the complex relationship between commuting and health by mainly focusing on the understanding of the commuting effects on workers’ health [6]. Commuting deteriorates commuters’ health through two separate channels, a quantitative one related to commuting time and a qualitative one related to the commuters’ ways of commuting.

A long commute is associated with a higher mortality risk [7], greater exposure to pollution [8], increased stress [9], exhaustion [10], sleep disorders [11] and lower satisfaction felt during socialising [12] and leisure [13]. For example, in France, the mean duration of one-way commuting time between the place of residence and the workplace increased from 38.3 min in 2005 to 44.9 min in 2015 [14].

Secondly, modes of commuting generate specific health problems. Physical activity has been more often observed among pedestrians [15], cyclists [16] and public transport users [17] than by car users. Pedestrians, cyclists and public transport users have a lower body mass index (BMI) than car drivers [18, 19]. In contrast, high stress was found among car drivers and low stress among active commuters i.e., pedestrians and cyclists [20, 21]. In addition, active commuters reported higher satisfaction’s levels than car drivers and public transport users [22]. Passive commuting i.e., car and public transports, is associated with a low perceived health among workers [2]. Self-perceived health is a widely used indicator in the literature [2, 23].

Commuting is not the only factor that affects CBW’s health. Higher incomes allow people to buy more or better goods (e.g. organic food), or to participate in sports or leisure activities with positive health benefits [24]. An income hypothesis can be suggested here and would imply that each additional euro of income would raise individual health. Between income and self-perceived health, a positive association was highlighted [25]. In contrast, a negative relationship exists between income and BMI [26, 27] and also between income and health problems such as asthma, hearing problems and dental symptoms [28]. In the same vein, low income is correlated with low self-perceived health [29], more activity limitation [30], more chronic diseases [31], more disability [32] and lower participation in sports [33].

As each member state of the UE maintains its own social security national institutions, information about CBWs remains piecemeal [34]. De facto*,* widespread international datasets commonly used to analyse workers’ health, such as the European Working Conditions Survey (EWCS), the European Health Interview Survey (EHIS) or the EU Statistics on Income and Living Conditions (EU-SILC), are unusable in this case owing to the impossibility to identify CBWs. Since 1950, the labour survey (‘*Enquête Emploi*’) driven by the National Institute of Statistics and Economics Studies *(‘Institut National de la Statistique et des Etudes Economiques’* - INSEE) constitutes the main source of information on the French labour market and workforce [35]. Based on the European Union Labour Force Survey (EU LFS), the labour survey section ‘*Enquête Emploi*’ was the only dataset allowing researchers to identify and to better understand the commuting behaviours of CBWs.

The relationships between commuting abroad for work, health outcomes and income are under-documented. Research works are still needed that aim to identify if some groups are at more risk for ill health than others [2]. Our study aims to: (1) measure the prevalence for the perceived health status and the physical health factors (such as activity limitation, chronic diseases, disability and no leisure activities), (2) analyse their associations with commuting status as well as (3) with income and health index among CBWs.

## Methods

### Selection criteria

Between 2013 and 2018, 2,546,802 persons were surveyed in a repeated-cross sectional survey conducted with a representative sample of the French population. The population is made up of all the members (aged 15 or more) in a given household. Households are identified through the comprehensive housing-tax registers and invited to participate in the survey by post. The sample of households is stratified in order to be representative; many criteria are used, the most important are the region and the spatial type (urban center, suburban rings, multi-polarised municipalities, rural municipalities). The questionnaire [36] was administered face to face (for the first and the final measurement) and by telephone (for all other measurements). Since we cannot determine the number of people contacted who fulfilled our inclusion criteria in this dataset, the response rate cannot be calculated. Nevertheless, the INSEE indicates a response rate of 80%, and we have no reason to believe that our response rate is different.

**Fig. 1:**
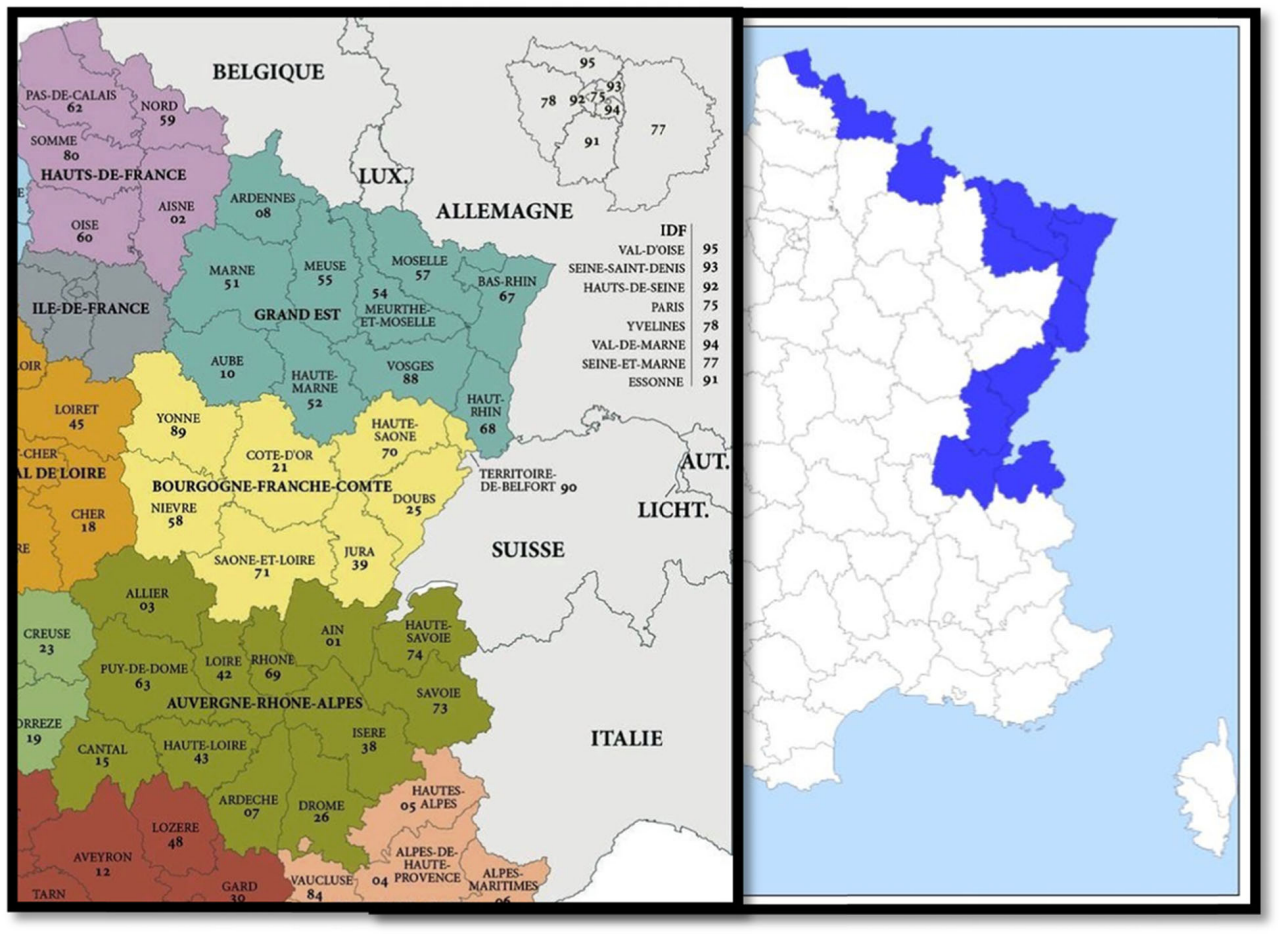
The French cross-border region. Source: Document complied by the authors with the help of the websites: http://www.Lion1906.com and https://www.cartes-2-france.com/cartographie/carte-france/x2-carte-france-regions-hd.jpg. The French cross-border region is composed of 11 departments: Ain, Ardennes, Doubs, Jura, Meurthe-et-Moselle, Moselle, Nord, Bas-Rhin, Haut-Rhin, Haute-Savoie and Territoire de Belfort. Of the commuters to Germany, 57 lived in Moselle, 143 in the Bas-Rhin, and 33 in the Haut-Rhin. Of those commuting to Belgium, 43 resided in the Ardennes, 31 in Meurthe-et-Moselle and 205 in Nord. Of the commuters to Switzerland, 70 lived in Ain, 250 in Doubs, 52 in Jura, 337 in Haut-Rhin, 554 in Haute-Savoie and 53 in Territoire de Belfort. Of the commuters to Luxembourg, 320 resided in Meurthe-et-Moselle and 287 in Moselle. The departments Meuse and Aisne did not fit the selection criteria and were therefore not included in the analyses

A sample of workers characterised by different workplace locations was extracted from the labour survey. Our sample fulfilled the following inclusion criteria: aged between 20 and 60 years, employed workers according to the International Labour Office (ILO) definition and residing in the French territory within the French cross-border departments (see Fig. 1: The French cross-border regions.). The French state is decomposed into three administrative districts: the *‘Communes’* (town), the *‘Départements’* (county) and the *‘Régions’* (states) (UK regions) (there are 34,970 *Communes*, 101 *Départements (called departments from now on)* and 13 *Régions)*. Using the ‘*Enquête Emploi*’ dataset, we retained areas in which CBWs are concentrated (i.e. nearby the border), while calculating the number and the share of CBWs per departments. All departments in which at least 50 CBWs lived and in which CBWs represent at least 1% of the workforce were included in the analyses. Four commute destinations were retained, namely Germany (DE), Belgium (BE), Switzerland (CH) and Luxembourg (LU), since these countries attracted 92% of the French’s CBWs [37]. To ensure the consistency and the replicability of the results, the first interrogation of each worker was retained whereas the last interrogation was used to ascertain the robustness of our findings. The first wave led to a larger sample than the sixth one, due to attrition (N1 = 22,828 vs. N6 = 19,506), leading us to favour the former over the latter. The selection criteria applied in order to obtain the final sample (*n* = 22,828) were:

1. Workers: who were currently in employment according to the ILO definition and settled in France, farmers and all workers who didn’t inform their wages were dropped and employees and individuals aged between 20 and 60 were retained.
2. Departments: departments in which at least 50 CBWs lived and in which their represented at least 1 % of the workforce and neighbouring departments with Germany, Belgium, Switzerland and Luxembourg were kept.
3. Data: Only the first interrogation was kept for each worker and missing values for the number of persons working at the local unit, overtime, sector and education were dropped.

### Data collection

Since 2013, the survey integrated information about workers’ health, and the module assessing this is composed of four questions (see health variables). Between 2013 and 2018, the number of CBWs per year (e.g. 404 for 2015) leads to combine the labour survey individual folders for years and the health questions. The labour survey benefits from the approval of the Institutional Review Board called *‘Comité du Label de la Statistique Publique’*, which depends on the *‘Conseil National de l’Information Statistique’* (CNIL). The questionnaire of the mother study has been previously published [36] and only the relevant questions were kept. Two parameters were considered to describe the worker’s situation, his commuting status and his country of destination. A worker can decide either to work in France or to commute abroad. CBWs are referred as workers that are currently working abroad, either in Belgium, Germany, Luxembourg or Switzerland and presently dwelling in France. Four questions assessing health outcomes were included in the labour survey and one question not directly linked to health issues was used to generate a variable relating to leisure activity participation.

- *Low perceived health:* This subjective scale included one item: ‘How do you rate your overall health?’ and responses options were: (1) very good, (2) good, (3) fair, (4) poor, and (5) very poor, (6) refusal and (7) do not know. For the statistical analyses, the score was dichotomised: (3), (4) and (5) were coded ‘low self-perceived health’ following the recommended cut-off scores [24, 25]. (6) and (7) were coded as missing values.
- *Activity limitation:* ‘Have you experienced restrictions in performing activities that people typically do because of a health problem for at least six months?’. Response options were: (1) yes heavily restricted, (2) yes, limited but not strongly, (3) no, not limited at all, (4) refusal and (5) do not know. Responses were coded: (1) and (2) ‘limitations due to health reasons’ and response (3) was coded as ‘no limitations due to health reasons’. (4) and (5) were coded as missing values.
- *Chronic diseases:* ‘Do you have an illness or health problem that is chronic or of a lasting nature?’ Responses options were: (1) yes, (2) no, (3) refusal and (4) do not know. Responses were coded: 1) ‘having a chronic disease’ vs. 2) ‘no chronic disease’. Responses 3) and 4) were coded as missing values.
- *Disability:* ‘Is your disability or loss of autonomy recognised by the administration?’ Response options were: (1) yes, (2) demand under review, (3) no, (4) refusal and (5) do not know. Responses were coded: (1) and (2) ‘handicapped’ vs. (3) ‘not handicapped’. Responses (4) and (5) were coded as missing values.
- *No leisure activities:* ‘During the past three months, did you take sports lessons or courses related to cultural or leisure activities?’ Response options were (1) yes and (2) no. Responses were coded: (2) ‘no physical or cultural activities’ vs. (1) ‘physical or cultural activities’.

The study included two sets of covariates: the demographic background (Var.1-Var.10) and the labour status (Var.11-Var.17) of the workers (see Table 1). Consolidated variables are those composed by the respondents’ answers to several questions.

**Table 1:** Covariates: demographic background and labour status variables

**Demographic background**	
(Var.1) ***Sex***: (2 categories: men vs. women)	
(Var.2) ***Age***: (4 categories: 20–29, 30–39, 40–49, 50–60)	
(Var.3) ***Education***: (3 categories: up to secondary school, up to Bachelor’s degree, Master’s degree & above)	
(Var.4) ***Occupational category***: (4 categories: white collars, intermediate professions, employees, blue collars)	
(Var.5) ***Father’s occupational category***: (7 categories: not filled out; farmers; artisans, merchants, company directors; white collars; intermediate professions; employees; blue collars) Assessed at the end of the respondent’s own schooling.	
(Var.6) ***Born abroad***: (2 categories: born in France vs. born abroad) Respondents were asked ‘Where were you born?’. Responses options were 1) in France and 2) not in France.	
(Var.7) ***Cohabiting***: (2 categories: living together with someone vs. living alone) Respondents were asked ‘Are you living together with someone in one household?’. Response options were (1) yes and (2) no. The variable cohabiting was preferred to the marital status i.e., the legal recognition of cohabitation, because it allows us to capture all the workers that beneficiated from a lower mortality rate [38] and not only those having the legal recognition of their situations.	
(Var.8) ***Children***: (2 categories: has a child (ren) vs. does not have child (ren)) Respondents were asked ‘Do you have children in the household or in alternate care?’. Response options were (1) yes and (2) no.	
(Var.9) ***Departments****:* (11 categories: departments of residence (see Fig. 1: The French cross-border regions))	
(Var.10) ***Urban area****:* (2 categories: place of residence located in an urban area with fewer than 200,000 inhabitants vs. with 200,000 inhabitants or more)	
(Var.11) ***Permanency of the job***: (3 categories: permanent contract, temporary contract, interim contract)	
**Labour status**	
(Var.12) ***Sector****:* (10 categories: not filled out; agriculture; industry-construction; trade-transport-accommodation and catering; information and communication; finance and insurance; real estate; scientific activities and administrative services; public administration; other services)	
(Var.13) ***Number of persons working at the local unit***: (5 categories: not filled out, 1 to 9 workers, 10 to 49 workers, 50 to 499 workers, 500 workers and more). Respondents were asked ‘How many employees are approximately on the site which employs you?’.	
(Var.14) ***Wage***: (3 categories: up to 2000 net € per month, premiums included, between 2001 and 4000€, 4001€ and higher) Nominal wage.	
(Var.15) ***Full-time/part-time employment***: (2 categories: full-time employment vs. part-time employment)	
(Var.16) ***Overtime***: (2 categories: overtime vs. no overtime) Respondents were asked ‘How many extra hours do you usually work per week in your professional job?’.	
(Var.17) ***Night work***: (2 categories: night work vs. no night work). Respondents were asked ‘Did you work during the night (between midnight and 5 a.m.) during the four precedent weeks?’.	

Sex, age, education, occupational category, father’s occupational category, departments, urban area, permanency of the job, sector, number of persons working at the local unit, wage and full-time/part-time employment are consolidated variables. As missing values were dropped, all variables are fully informed for each worker.

### Statistical analysis

To distinguish CBW from NCBW, a dichotomous variable was generated as well as four binary variables, one for each country of destination. Example, the variable for Germany was coded as: 1) is working in Germany and 0) is working in France but lives in the same department, as CBW working in Germany. Consequently, from this coding, each group of NCBWs is defined by a single combination of departments. For example, commuters to Germany (DE CBWs) and their non-commuter counterparts (DE NCBWs) dwelled in Moselle, Bas-Rhin and Haut-Rhin. However, a given department can host the commuters and their non-commuter counterparts toward different countries. For example, both commuters toward Belgium and Luxembourg and their non-commuter counterparts dwelled in Moselle. This allows considering the local health features, while taking the closest possible counterparts of the CBWs. A summary of the place of residence by commuting status and country of destination is provided (see Additional files, Table A1). To preselect the covariates, chi-square tests were used for qualitative variables and Student’s t-tests for the quantitative ones, including those which were significant (*p* < 0.10) for at least one of the health outcomes. The covariates were selected based on the previously described theoretical frameworks and introduced in three steps. A logistic model was estimated for each health variables and covariates were introduced in order to predict the probability of CBWs to report a health issue compared to NCBWs.

In a first step, only the commuting status was introduced in the model (unadjusted model). In a second step, the demographic background of the workers was introduced in the adjusted model. In a third step, the labour status of the workers was introduced in the fully adjusted model. All the outcome variables were coded with the aim to model the probability of being in ill health according to the commuting status. An odds-ratio greater than one can also be understood as verification of ill health for CBWs compared to NCBWs, whereas an odds-ratio smaller than one indicated a better health in the group of CBWs compared to the reference group of NCBWs.

The Health Index (5 items) was an additional measure obtained with the score of four physical health variables and the perceived health score and calculated with values between 0 and 5: ‘the higher the score, the healthier the worker’. For each item, one point was assigned if the answer indicated a high health state and no point if the answer indicated a poor health. For example, the absence of disability was considered as an indication of a good health state. For the perceived health, one point was assigned if the respondents estimated their health as ‘very good’ or ‘good’, whereas a zero score was assigned if the answer was ‘fair’, ‘poor’ or ‘very poor’. For each worker, scores of the five items were aggregated. A linear model was used to investigate the relationship between income and health index. An interaction between the commuting status and the health index was introduced in the linear model to predict which wage is needed to reach a certain unit of the health index. Binary logistic regression modelling was used to determine associations between the commuting status and each of the five health outcomes. Odds ratios were estimated with a 5% risk of error i.e., 95% confidence intervals (CI). Covariates were added to the model to provide adjusted and fully adjusted associations for CBWs as a whole, as well as for CBWs of the different country destinations. All statistical analyses were performed using the software STATA 13.0.

## Results

For the analysis, 22,828 observations (2456 CBWs vs. 20,372 NCBWs) were retained. The sample consisted of 8 groups of workers from 4 countries of destination: for example, CBWs working in Germany (*n* = 233) and NCBWs working in France but living in the same departments as the CBWs to Germany (*n* = 6895). CBWs were more often men, blue collars, born abroad, employed under permanent contract, in the industry and the construction, in large companies, working at night and higher wages than the NCBWs (see Table 2). Commuters to Germany were of a special interest. Male, low-skilled and blue collar workers were overrepresented. They were the oldest group of CBWs, more often employed in full-time jobs, in the industry and construction sector, in large companies and worked overtime more than other CBWs.

**Table 2:** Demographic background and labour status by commuting status and countries of destination. (%, mean years and Euros)

	DE NCBWs	DE CBWs	***p***^**1**^	BE NCBWs	BE CBWs	***p***^**1**^	CH NCBWs	CH CBWs	***p***^**1**^	LU NCBWs	LU CBWs	***p***^**1**^	Total NCBWs	Total CBWs	***p***^**1**^
**Adjustment variables**: **Demographic background**															
Sex: Male (%)	51	66	**	51	64	*	49	64	***	51	67	***	**51**	**65**	***
Age (Mean years)	41	46	***	40	39	NS	41	40	NS	41	39	***	**41**	**41**	**NS**
Education: Up to secondary education (%)	64	70	NS	59	71	***	65	57	***	63	66	NS	**62**	**62**	**NS**
Occupational category: Blue collar (%)	28	47	**	23	55	***	27	31	NS	26	38	**	**25**	**37**	***
Father’s occupational category: Blue collar (%)	43	49	NS	42	51	**	39	38	NS	45	51	NS	**41**	**43**	**NS**
Born abroad (%)	11	26	***	6	19	***	9	18	***	9	13	**	**8**	**18**	***
Cohabiting (%)	67	76	NS	69	72	NS	71	71	**	67	68	NS	**69**	**71**	**NS**
Children (%)	49	41	NS	52	61	**	53	53	*	50	55	NS	**52**	**53**	**NS**
Urban area (%)	39	18	***	51	43	**	10	6	***	42	20	***	**34**	**14**	***
**Full adjustment variables: Labour status**															
Permanency of the job: Permanent contract (%)	87	89	NS	85	84	NS	88	94	***	86	90	*	**87**	**92**	***
Sector: Industry & construction (%)	24	57	***	19	47	***	27	39	***	21	34	***	**23**	**40**	***
Number of persons working at the local unit: > = 500 workers (%)	17	33	***	17	16	*	12	22	***	14	21	***	**15**	**22**	***
Wage (Mean €)	1817	2789	***	1808	2153	***	1791	4027	***	1835	2558	***	**1814**	**3383**	***
Full-time/part-time employment: Full-time employment (%)	82	90	**	81	90	**	82	77	**	81	86	**	**82**	**82**	**NS**
Overtime (%)	26	34	***	25	19	NS	28	31	NS	26	23	NS	**26**	**28**	**NS**
Night work: yes (%)	12	15	NS	11	22	NS	10	11	NS	13	20	**	**11**	**14**	*
**N**	**6895**	**233**		**8304**	**279**		**6941**	**1316**		**3828**	**607**		**20372**	**2456**	

DE NCBWs: Non-commuters toward Germany. Are working and dwelling in France

DE CBWs: Cross-border workers toward Germany. Are working in Germany and dwelling in France

BE NCBWs: Non-commuters toward Belgium. Are working and dwelling in France

BE CBWs: Cross-border workers toward Belgium. Are working in Belgium and dwelling in France

CH NCBWs: Non-commuters toward Switzerland. Are working and dwelling in France

CH CBWs: Cross-border workers toward Switzerland. Are working in Switzerland and dwelling in France

LU NCBWs: Non-commuters toward Luxembourg. Are working and dwelling in France

LU CBWs: Cross-border workers toward Luxembourg. Are working in Luxembourg and dwelling in France

*p*^**1**^: significance p* ≤ 0.1 | p** ≤ 0.05 | p*** ≤ 0.01; chi-square test for qualitative variables and Student’s t-test for quantitative variables; significance level of the difference between the total NCBWs and the total CBWs. Aggregation of contingency tables 2 × 2 with 1 degree of freedom, except for age and wage

CBWs declared less often than NCBWs: low self-perceived health, activity limitation, chronic diseases and disability (see Table 3). CBWs do not differ from NCBWs regarding no leisure activities. Distinguishing characteristics of commuters to Germany were a higher prevalence of low perceived health, limitations, chronic diseases and with the lower prevalence of no leisure activities. In contrast, commuters to Switzerland reported the lowest prevalence of low perceived health, activity limitation and disability, whereas commuters to Luxembourg had the lowest prevalence of activity limitation and chronic diseases and the highest prevalence of no leisure activities. Commuters toward Belgium declared the lowest prevalence of disability as well. Commuters toward Germany and Belgium have no health outcomes significantly different from their NCBW counterparts, at the opposite of commuters toward Luxembourg and Switzerland.

**Table 3:** Descriptive values of health outcomes by countries of destination.  (%)

	**DE NCBWs**	**DE CBWs**	***p***^1^	**BE NCBWs**	**BE CBWs**	***p***^1^	**CH NCBWs**	**CH CBWs**	***p***^*1*^
Low perceived health	18	22	NS	17	15	NS	16	12	***
Activity limitation	13	17	NS	13	11	NS	13	9	***
Chronic diseases	24	33	NS	23	19	NS	23	19	*
Disability	5	3	NS	5	2	NS	5	2	***
No leisure activities	85	81	NS	83	88	NS	83	82	NS
	**LU NCBWs**	**LU CBWs**	***p***^1^	**Total NCBWs**	**Total CBWs**	***p***^1^			
Low perceived health	17	13	***	**17**	**13**	***			
Activity limitation	14	9	***	**13**	**10**	***			
Chronic diseases	26	17	***	**23**	**20**	***			
Disability	5	3	***	**5**	**2**	***			
No leisure activities	86	89	*	**83**	**84**	**NS**			

*p*^**1**^: significance p* ≤ 0.1 | p** ≤ 0.05 | p*** ≤ 0.01 chi-square test for the difference between total NCBWs and total CBWs. Aggregation of contingency tables 2 × 2 with 1 degree of freedom

From the annual Health at a Glance reports, published by the OECD and covering the periods of the survey, the high perceived health indicators of the countries and the OECD indicators were reported to compare them with the different prevalence rates obtained by each group (see Additional files, Table A2). CBWs expressed a higher perceived health than the population of their country of destination. We observed that all frequencies of the CBW groups are higher than the EU states’ indicators and the OECD indicators. Considering the latter, 65% of the German citizens expressed a high perceived health against 78% for CBWs toward Germany. The same pattern was found for Belgium, Switzerland and Luxembourg with respectively 74% against 85, 81% against 88, 72% against 87%. Furthermore, Germans citizens reported the lowest share of high perceived health whereas Swiss citizens had the highest one with respectively 65 and 81%, which is consistent with our precedents results. Belgians and Luxembourgers citizens being in an intermediate position between these two extreme cases with respectively 74 and 72%.

For the fully adjusted model, the commuting status is significantly associated with health outcomes (see Table 4). We found a lower probability of CBW to report chronic diseases and disability compared to NCBW. These results suggest that CBWs are healthier than their NCBWs counterparts. However, no association was found between the commuting status and low perceived health, activity limitation and reporting no leisure activity. The full regression tables are available (see Additional files, Tables A3). Commuters to Germany were the only group of CBWs for which a negative association between the commuting status and no leisure activities i.e., the only workers who did not agree with the statement of no leisure activities, was found. Furthermore, they were the only group of CBWs for which a positive association with health outcomes was found. They had a higher likelihood to express chronic diseases than their NCBW counterparts, ascertaining their poorer health state previously observed. Commuters to Luxembourg were less likely to report activity limitation, chronic diseases and disability compared to their NCBW counterparts, suggesting that they were the healthiest group of CBWs in our sample. A negative association was found between the commuting status and disability for both commuters toward Belgium and Switzerland. After controlling for both demographic background and labour status variables it turned out that the better health outcomes of the commuters toward Switzerland were not significant, suggesting that their better health state might be due to these confounders, most probably to their higher wages.

**Table 4:** Associations between CBW status and health outcomes

NBCWs = reference group value 1										
Model of regression	DE CBWs	***p***^**1**^	BE CBWs	***p***^**1**^	CH CBWs	***p***^**1**^	LU CBWs	***p***^**1**^	Total CBWs	***p***^**1**^
**Unadjusted**										
Low perceived health	1.31 (0.934–1.846)	NS	0.89 (0.615–1.292)	NS	0.70 (0.576–0.845)	***	0.69 (0.532–0.907)	***	**0.75** **(0.660–0.858)**	***
Activity limitation	1.32 (0.912–1.903)	NS	0.79 (0.492–1.254)	NS	0.65 (0.515–0.825)	***	0.65 (0.481–0.870)	***	**0.73** **(0.622–0.850)**	***
Chronic diseases	1.52 (1.128–2.039)	***	0.81 (0.583–1.132)	NS	0.79 (0.668–0.944)	***	0.60 (0.471–0.753)	***	**0.82** **(0.731–0.925)**	***
Disability	0.58 (0.279–1.201)	NS	0.37 (0.159–0.861)	**	0.33 (0.197–0.547)	***	0.50 (0.313–0.811)	***	**0.40** **(0.299–0.537)**	***
No leisure activities	0.73 (0.513–1.044)	*	1.44 (0.976–2.123)	*	0.94 (0.766–1.140)	NS	1.43 (1.068–1.912)	**	**1.06** **(0.917–1.221)**	**NS**
**Adjusted**										
Low perceived health	1.10 (0.772–1.576)	NS	0.82 (0.550–1.209)	NS	0.74 (0.602–0.905)	***	0.72 (0.540–0.954)	**	**0.78** **(0.675–0.893)**	***
Activity limitation	1.04 (0.713–1.511)	NS	0.75 (0.456–1.228)	NS	0.71 (0.552–0.900)	***	0.64 (0.463–0.894)	***	**0.74** **(0.626–0.871)**	***
Chronic diseases	1.39 (1.020–1.886)	**	0.84 (0.592–1.180)	NS	0.80 (0.667–0.960)	**	0.66 (0.516–0.840)	***	**0.81** **(0.713–0.914)**	***
Disability	0.45 (0.217–0.947)	**	0.32 (0.135–0.747)	***	0.33 (0.199–0.550)	***	0.49 (0.296–0.818)	***	**0.39** **(0.290–0.529)**	***
No leisure activities	0.53 (0.358–0.782)	***	1.19 (0.798–1.766)	NS	0.87 (0.701–1.071)	NS	1.21 (0.892–1.634)	NS	**0.95** **(0.816–1.102)**	**NS**
**Fully adjusted**										
Low perceived health	1.18 (0.815–1.710)	NS	0.91 (0.606–1.361)	NS	0.90 (0.678–1.187)	NS	0.89 (0.658–1.215)	NS	**0.89** **(0.756–1.045)**	**NS**
Activity limitation	1.12 (0.756–1.660)	NS	0.83 (0.499–1.383)	NS	0.81 (0.602–1.085)	NS	0.67 (0.465–0.955)	**	**0.85** **(0.710–1.012)**	*
Chronic diseases	1.42 (1.035–1.953)	**	0.90 (0.634–1.287)	NS	0.95 (0.752–1.209)	NS	0.71 (0.543–0.924)	**	**0.87** **(0.752–0.996)**	**
Disability	0.53 (0.248–1.137)	NS	0.37 (0.156–0.876)	**	0.52 (0.273–0.978)	**	0.54 (0.303–0.969)	**	**0.48** **(0.342–0.672)**	***
No leisure activities	0.53 (0.355–0.791)	***	1.23 (0.822–1.841)	NS	0.92 (0.716–1.190)	NS	1.20 (0.872–1.658)	NS	**0.97** **(0.822–1.133)**	**NS**
**N**	**233**		**279**		**1316**		**607**		**2456**	

Unadjusted: commuting status

Adjusted: commuting status, sex, age, education, occupational category, father’s occupational category, born abroad, cohabiting, children, departments, urban area

Fully adjusted: commuting status, sex, age, education, occupational category, father’s occupational category, born abroad, cohabiting, children, departments, urban area, permanency of the job, sector, number of people employed at the local unit, wage, full-time/part-time employment, overtime, night work

*p*^**1**^: significance p* ≤ 0.1 | p** ≤ 0.05 | p*** ≤ 0.01; Wald test

For the whole sample of workers, a higher income is associated with a higher health index, since a wage premium of 104 euros (€) led to a marginal improvement in the health index (see Additional files, Table A4). Specifically by group of workers, a marginal improvement in the health index is correlated with a wage premium of 81€ for NCBWs and 161€ for CBWs. This positive association between health index and wage is highly significant for each group of workers. Since only 25 CBWs had a health index equal to 0 and the wide confident intervals, the prediction for this level of the health index might be considered as an outlier, confirming the positive relationship between wage and health index for both CBWs and CBWs.

## Discussion

Our research aimed to highlight health disparities between CBWs and NCBWs and in the specific CBW group. Our key findings are: (1) CBWs are healthier than NCBWs. (2) Stronger health disparities were found in the different CBWs’ groups from different work destinations with commuters to Luxembourg exhibiting the best health outcomes and those toward Germany the worst. (3) Based on these data, the spillover phenomenon assumption must be rejected. Our main findings between commuting status and higher health outcomes are in contrast to findings of previous studies [2, 9–11, 20, 21]. Importantly, our study pointed out a CBW paradox, because being a commuter constitutes a risk-free factor that protected workers against ill health. How is that possible?

The health gap between CBWs and NCBWs might be explained by the fact that CBWs perceived higher wages than NCBWs, since higher incomes have been associated with better health outcomes in the literature. Compared to NCBWs, CBWs have a better access to health-friendly consumption or activities (like purchasing organic food, seeking alternative medicine and participating in expensive sports). For both NCBWs and CBWs, the positive association between wage and health index is confirmed in our analysis. In the absence of CBWs’ self-selection or employers’ selection, we should expect that commuting will worsen CBWs’ health, but that their higher incomes will improve their health compared to NCBWs. We assumed that CBWs report a lower perceived health status, higher limitation of activities, a higher number of chronic diseases and disability, and less leisure activities than NCBWs. Nevertheless, even after controlling our results for wages, CBWs are still in better health than NCBWs, with our data exhibiting high estimated coefficients. Thus, this health gap can only be interpreted by the existence of a social selection processes.

The social selection of the workers can originate from either the side of the workers or the employers. The so-called healthy worker effect, a well-known phenomenon in epidemiology [39], was first observed among workers compared to the whole population [40]. The healthy commuter theory [2] argues that only the healthiest workers will undertake longer commutes whereas those affected by ill health will choose to reduce their commuting time in order to minimise stress, tiredness and dissatisfaction resulting from commuting. Our study emphasises this phenomenon among CBWs too, suggesting that only the healthiest workers make the decision to work in a neighbouring country. This could explain why, even after being affected by the negative consequences of commuting, they still report a better health state than NCBWs. That could be the case if the positive dynamic resulting from the self-selection process is stronger than the negative one of commuting on workers’ health. In this respect, our findings are consistent with the healthy commuter theory [2].

Likewise, health may play a role in the selection of CBWs by recruiters during the matching process in the country of destination. Because hiring can be considered as an investment into uncertainty, recruiters want to minimise risks during the hiring procedure. Recruiters anticipate that commuting is gruelling and consider health as a longevity capital upon the cross-border labour market. Another argument could be that due to the higher labour cost in the destination countries, employers might have more productivity expectations leading them to examine the candidates’ health status more closely. This could be especially the case in demanding business areas (like the industry, construction or catering), in which French commuters are overrepresented. Sociologists will argue that recruiters might be more demanding health-wise with CBWs because they are not considered to be a part of the national community in the country of destination but simply a production factor. Therefore, we can presume that recruiters carefully analyse minor health signals that candidates involuntarily share, and, for example, exclude candidates who exhibit difficulties in sitting down, a livid skin colour, tiredness, overweight [41] or physical disabilities [42].

CBWs compare two possible situations: (1) working in their country of residence (less commuting and lower wages) or (2) working abroad (more commuting and higher wages). Let us suppose that the net gain from cross-border mobility (G) is equal to the wage gap between the country of destination and the country of residence (W), minus the temporal and health consequences of the mobility (C). Then, workers with a poorer health status than the average will face higher mobility cost and, thus, will obtain a smaller net gain from their mobility. For example, handicapped workers in wheelchairs will need more time to commute the same distance as non-disabled workers, leading to a higher mobility cost and finally to a smaller net profit. In other words, disability might be interpreted as a disincentive to commute abroad. In this respect, health is defined as a major driver of the cross-border mobility. We can assume that workers have the capacity to estimate their resilience regarding a particular professional context, and this estimation might be a parameter in the decision to work abroad. Such an ability has already been observed among workers. For example, candidates for shiftwork had more compatible sleep behaviours than those who applied for day work [43].

Secondly, major health disparities arose within the groups of CBWs, regarding the country of destination. Our analyses revealed that commuters to Luxembourg are the healthiest workers in our sample whereas those to Germany are the commuters with the poorest health state. How can we explain health disparities among CBWs?

Let us contextualise our findings concerning the main health disparities that arose within the groups of CBWs regarding the countries of destination. Commuters to Germany single out themselves since they are the only group of CBWs reporting less no leisure activities than the NCBWs. Physical activity is higher in Germany than in France [44], which could indicate the existence of stronger sport-friendly norms in German society. A possible explanation might be that CBWs are socialised into the countries of destination, appropriating themselves some of the local norms and values operative in the foreign societies. This socialisation is even more possible knowing that three-quarters of the French workers commuting to Germany are living in the Alsace (see Fig. 1: The French cross-border regions), a French area strongly influenced by German culture as is apparent in the dialect, the cuisine, the architecture, or even in the names of the villages (Alsace was a *Reichland* (Imperial territory) from 1871 to 1918 and occupied by the Nazis from 1940 to 1944). Commuters to Germany might have more chronic diseases since they are the oldest group of CBWs, are more employed in the industry and the construction or are more often working overtime. However, since associations were controlled for these variables, no explanation has been found. Commuters to Luxembourg might be the healthiest CBWs because they are the younger group of CBWs. However, as a control was introduced in the model for age, no explanation has been found.

We must remark that when we controlled for demographic background variables, estimated odds-ratios (OR) decreased slightly, indicating that health inequalities between workers had less to do with workers’ demographic background. The introduction of the labour status entailed a major reduction of the OR for all health outcomes, which may indicate the important contribution of the labour status in health inequalities among workers.

Finally, to start this paper we assumed that CBWs are enduring indirect consequences of their specific lifestyle on their health. Because CBWs commute longer than NCBWs to their place of employment, we expected that their professional lifestyle reduces their free time. As a consequence, the supplementary time spent in traffic cannot be invested in health-friendly activities (such as sport, meditation, socialising) leading them to report more no leisure activities than the NCBWs. Although a spillover phenomenon has been highlighted for commuters [2], our results lead us to reject such an assumption for the CBWs.

### Strengths and limitations

One strength point of our study is the large sample size, which makes the analysis of the association between commuting status and health factors possible. More importantly, our study design avoids possible bias resulting from different demographic background and labour statuses, which may explain morbidity differences between workers [39]. Controlling results with 17 demographic background and labour status variables may seriously reduce these biases in the statistical analysis. Furthermore, no subjects were included twice in our sample, since we only retained the first interrogation of each worker. The use of the labour survey excludes the risk of representative bias, considering that only a representative part of the population was investigated. The demographic background of the commuters in our sample is consistent with the commuter profiles found in other studies [34, 45].

Controlling for demographic background variables, like department and urban area, aid to avoid a local selection bias. Indeed, some areas might have better health conditions than other areas, due to an easier access to health care or a higher diagnostic quality (resulting from newer equipment or better medical training) explaining health differences between workers [46]. Our results confirmed that indicators of labour status (permanency of the job, sector, number of persons working at the local unit, wage, full-time/part-time employment, overtime and night work) need to be considered in the analysis as a main source of health inequalities among workers. For example, it has been shown that workers in large companies have better access to health care, which can constitute a protective factor against diseases [47]. Furthermore, adjusting for overtime or night work allows separating the association between commuting status and health from other work-related choices [2].

As a validation process of the relevance of our exposure variable, the commuting status, we summarised (see Additional files, Table A5) the coefficients significance and the mean coefficient values in order to determine which variables are the most associated with health outcomes (except no leisure activities) for the fully adjusted model. We only retained variables for which the significant summation is greater than two, meaning that coefficients are significant for at least two health variables, and we only displayed the mean coefficient values for these retained variables. As expected, age is positively associated with health limitations and is the variable the more strongly associated with health outcomes. A healthy worker portrait can be outlined from these results: CBW, young, educated, not blue collar, cohabiting, having children, in interim, working in the industry & construction, in trade, transport, lodging & catering, in scientific and technical activities, not working in local unit of more than 50 workers, earning a high wage, working full-time and at night and no overtime. Our results stressed the importance of the commuting status in health inequalities among workers, since this variable had an estimated coefficient of the same magnitude as education and wage. The commuting status is to be considered of similar importance as other ‘heavy variables’ like major demographic background and labour status variables. Furthermore, consistent results with our findings were found when workers’ last interrogation was retained for the statistical analysis (see Additional files, Table A6).

One of the limitations of our study is probably its ‘French focus’. Even if our study might have an international scope because of the spread of French workers in four different countries, our findings cannot be generalised without complementary studies on this topic from other countries. As well, the dataset did not include the lifestyle history of the workers, like smoking or drinking alcohol, which are associated with poorer health outcomes. Finally, our estimates might have underestimated the health gap between the two groups, since a control for wage was introduced in the regression.

## Conclusion: policy perspectives

As a consequence, the newly founded European Labour Authority (ELA) should take into account health policies as a promising way to support the cross-border mobility among countries of the European Union.

Firstly, our study highlighted a free rider phenomenon among European countries. Some countries are using the healthiest workforce of the surrounding countries, without having to bear health expenditures of other workers, or for the inactive part of the population stayed behind the border. The countries where CBWs are employed only compensate the health expenditures of the CBWs. As CBWs and their employers pay their social contribution in the country of employment, this should represent a deadweight loss for the country of residence, since healthy workers should pay for those who are in poorer health. The creation of a European social security system might solve this issue, thus making the benefits of a healthy and mobile workforce shared by all European countries.

Secondly, the European principle of free movement of workers grants EU citizens the ‘right to work in another member state’ [48] whereas in reality, only the healthiest workers commute to other countries. Health disparities among individuals have created a differentiated access to the cross-border labour market, leading to the generation of economic inequalities within the EU. This situation is questioning the *isonomia* principle of the European law, which might feed the anti-EU feeling across the population, in a tense context marked by the awakening of populism and the disruption of EU values. As a consequence, to reduce economic inequalities, a health policy aiming to compensate health disparities is recommended. Several potential pathways are practicable: (1) An awareness campaign about cross-border mobility targeting sick people might be useful to increase the cross-border flows of workers among the EU. This campaign could be reinforced by a support programme provided by the national employment agencies. (2) The establishment of a labour organisation more compatible with the ‘sickness career’ [49] for workers in ill health. (3) Decreasing the mobility cost of sick workers will increase the net benefit of the commuting decision as described above, and thus generate more incentives to commute. Specifically, building new car parks, establishing special traffic lanes, or the creation of a free mobility programme in the public transport system for sick workers could be efficient in this respect.

## Supplementary Information

**Additional file 1: Dataset.** Dataset used in the mother study, the French part of the Labour Force Survey for years 2013–2018. **Table A1**: Workers’ departments of residence by commuting status and country of destination.%. **Table A2**: Good/very good perceived health status and countries’ indicators in 2013, 2015, and 2017.**Tables A3**: Associations between health limitations and demographic background and labour status variables. **Table A4**: Predicted wages in €, by health index units. **Table A5**: Associations between health limitations demographic background and labour status variables: summary table. **Table A6**: Associations between commuting status and health outcomes. (Last interrogation).

## Data Availability

Data supporting our findings can be found in the (see Additional files). The questionnaire was not produced by the authors.

## Abbreviations

CBWs: Cross-border workers
NCBWs: Non cross-border workers
ELA: European Labour Authority
ILO: International Labour Office
