# Supplementary material for: Cross-border mobility in European countries: associations between cross-border worker status and health outcomes
Source: BMC Public Health. 2021 Mar 24;21:588. doi: 10.1186/s12889-021-10564-8 (PMC7992783; doi:10.1186/s12889-021-10564-8)
Supplement: Supplementary file 1 — Additional file 1: Dataset. Dataset used in the mother study, the French part of the Labour Force Survey for years 2013–2018. Table A1: Workers’ departments of residence by commuting status and country of destination.%. Table A2: Good/very good perceived health status and countries’ indicators in 2013, 2015, and 2017.Tables A3: Associations between health limitations and demographic background and labour status variables. Table A4: Predicted wages in €, by health index units. Table A5: Associations between health limitations demographic background and labour status variables: summary table. Table A6: Associations between commuting status and health outcomes. (Last interrogation). [file 12889_2021_10564_MOESM1_ESM.docx]

**ADDITIONAL FILES:**

**Dataset**

Enquête Emploi en continu (version FPR) - 2018, INSEE [producteur], ADISP [diffuseur]

Enquête Emploi en continu (version FPR) - 2017, INSEE [producteur], ADISP [diffuseur]

Enquête Emploi en continu (version FPR) - 2016, INSEE [producteur], ADISP [diffuseur]

Enquête Emploi en continu (version FPR) - 2015, INSEE [producteur], ADISP [diffuseur]

Enquête Emploi en continu (version FPR) - 2014, INSEE [producteur], ADISP [diffuseur]

Enquête Emploi en continu (version FPR) - 2013, INSEE [producteur], ADISP [diffuseur]

**Table A1: Workers’ departments of residence by commuting status and country of destination. %.**

|  | **DE NCBWs** | **DE CBWs** | **BE NCBWs** | **BE CBWs** | **CH NCBWs** | **CH CBWs** | **LU NCBWs** | **LU CBWs** | **Total NCBWs** | **Total CBWs** |
| --- | --- | --- | --- | --- | --- | --- | --- | --- | --- | --- |
| Ain (01) |  |  |  |  | 19 | 5 |  |  | 7 | 3 |
| Ardennes (08) |  |  | 11 | 15 |  |  |  |  | 4 | 2 |
| Doubs (25) |  |  |  |  | 20 | 19 |  |  | 7 | 10 |
| Jura (39) |  |  |  |  | 11 | 4 |  |  | 4 | 2 |
| Meurthe-et-Moselle (54) |  |  | 19 | 11 |  |  | 42 | 53 | 8 | 14 |
| Moselle (57) | 32 | 24 |  |  |  |  | 58 | 47 | 11 | 14 |
| Nord (59) |  |  | 70 | 74 |  |  |  |  | 29 | 8 |
| Bas-Rhin (67) | 42 | 61 |  |  |  |  |  |  | 14 | 6 |
| Haut-Rhin (68) | 26 | 14 |  |  | 25 | 26 |  |  | 9 | 15 |
| Haute-Savoie (74) |  |  |  |  | 20 | 42 |  |  | 7 | 23 |
| Territoire-de-Belfort (90) |  |  |  |  | 4 | 4 |  |  | 1 | 2 |
| **N** | **6,895** | **233** | **8,304** | **279** | **6,941** | **1,316** | **5,868** | **849** | **20,372** | **2,456** |

**Table A2: Good/very good perceived health status and countries’ indicators in 2013, 2015, and 2017.**

| **Good and very good** | **DE**  **NCBWs** | **DE CBWs** | **BE NCBWs** | **BE CBWs** | **CH NCBWs** | **CH CBWs** | **LU NCBWs** | **LU CBWs** | **Total NCBWs** | **Total CBWs** |
| --- | --- | --- | --- | --- | --- | --- | --- | --- | --- | --- |
| High perceived health | 82 | 78 | 83 | 85 | 84 | 88 | 83 | 87 | **83** | **87** |
|  | **DE** | **DE** | **BE** | **BE** | **CH** | **CH** | **LU** | **LU** | **France** | **OCDE** |
| Indicators 2017* | 65 | | 74 | | 81 | | 72 | | **67** | **69** |
| Indicators 2015* | 65 | | 75 | | 79 | | 70 | | **68** | **68** |
| Indicators 2013 * | 65 | | 74 | | 80 | | 71 | |  |  |

**Health at Glance reports: 2015, 2017, 2019; OECD (2015, 2017, 2019), Health at a Glance 2015, 2017, 2019: OECD Indicators, OECD Publishing, Paris.*

*[*[*http://dx.doi.org/10.1787/health_glance-2015en*](http://dx.doi.org/10.1787/health_glance-2015en)*;* [*http://dx.doi.org/10.1787/health_glance-2017-en*](http://dx.doi.org/10.1787/health_glance-2017-en)*;* [*https://doi.org/10.1787/4dd50c09-en*](https://doi.org/10.1787/4dd50c09-en)*]*

**Tables A3: Associations between health limitations and demographic background and labour status variables**

| **Low perceived health** | **Unadjusted** |  | **Adjusted** |  | **Fully adjusted** |  |
| --- | --- | --- | --- | --- | --- | --- |
| **Variables** | **OR** | **95%CI** | **OR** | **95%CI** | **OR** | **95%CI** |
| **Commuting status**  NCBW  CBW | Ref  0.75*** | 0.66 – 0.86 | Ref  0.78*** | 0.68 – 0.89 | Ref  0.89 | 0.76 – 1.05 |
| **Sex**  Women  Men |  |  | Ref  0.87*** | 0.80 – 0.96 | Ref  1.01 | 0.92 – 1.11 |
| **Age**  20-29  30-39  40-49  50-60 |  |  | Ref  1.77***  2.91***  4.49*** | 1.49 – 2.11  2.48 – 3.42  3.86 – 5.23 | Ref  1.81***  3.04***  4.78*** | 1.52 – 2.16  2.56 – 3.60  4.06 – 5.63 |
| **Education**  Up to secondary school  Up to Bachelor’s degree  Master’s degree & above |  |  | Ref  0.72***  0.69*** | 0.64 – 0.81  0.56 – 0.85 | Ref  0.74***  0.72*** | 0.65 – 0.83  0.59 – 0.89 |
| **Occupational category**  Blue collars  Employees  Intermediates professions  White collars |  |  | 1.36***  1.18***  Ref  0.85* | 1.21 – 1.53  1.05 – 1.33  0.72 – 1.01 | 1.29***  1.09  Ref  0.95 | 1.13 – 1.46  0.97 – 1.24  0.81 – 1.13 |
| **Father’s occupational category**  Not field out  Farmers  Artisans, merchants, company directors  White collars  Intermediates professions  Employees  Blue collars |  |  | 1.12  0.89  0.91  0.96  Ref  0.95  0.93 | 0.95 – 1.33  0.71 – 1.13  0.77 – 1.09  0.80 – 1.16  0.79 – 1.13  0.83 - 1.06 | 1.12  0.88  0.92  0.95  Ref  0.95  0.93 | 0.95 – 1.33  0.70 – 1.11  0.78 – 1.10  0.79 – 1.14  0.80 – 1.14  0.82 - 1.05 |
| **Born abroad**  Born in France  Not born in France |  |  | Ref  1.18*** | 1.04 – 1.35 | Ref  1.17** | 1.03 – 1.33 |
| **Cohabiting**  Alone  Couple |  |  | Ref  0.85*** | 0.77 – 0.93 | Ref  0.84*** | 0.77 – 0.93 |
| **Children**  No children  Children |  |  | Ref  0.91** | 0.83 – 0.99 | Ref  0.90** | 0.82 – 0.98 |
| **Department**  Ain 01  Meurthe-et-Moselle 54  Moselle 57  Haut-Rhin 68  Haute-Savoie 74  Territoire de Belfort 90 |  |  | Ref  1.14  1.11  1.10  1.02  1.38* | 0.93 – 1.40  0.91 – 1.35  0.89 – 1.35  0.82 – 1.27  0.99 – 1.92 | Ref  1.14  1.13  1.13  1.05  1.38* | 0.93 – 1.41  0.92 – 1.37  0.91 – 1.39  0.84 – 1.30  0.99 – 1.93 |
| **Urban area**  Rural area  Urban area |  |  | Ref  1.07 | 0.97 – 1.19 | Ref  1.06 | 0.96 – 1.18 |
| **Permanency of the job**  Open ended contract  Fix-term contract  Interim |  |  |  |  | Ref  0.95  0.95 | 0.81 – 1.12  0.71 – 1.26 |
| **Sector**  Not filled out  Agriculture  Industry & construction  Trade, transport, lodging & catering  Information & communication  Finance & insurance  Real estate  Scientific & technical activities  Public administration  Other services |  |  |  |  | 0.67  1.10  1.00  1.02  1.16  0.97  0.77  0.99  Ref  0.96 | 0.33 – 1.38  0.71 – 1.71  0.88 – 1.14  0.90 – 1.15  0.79 – 1.71  0.75 – 1.24  0.52 – 1.13  0.84 – 1.17  0.74 – 1.24 |
| **Number of persons working at the local unit**  Not filled out  1-9  10-49  50-499  500+ |  |  |  |  | 1.09  Ref  1.16**  1.19**  1.10 | 0.89 – 1.33  1.01 – 1.32  1.04 – 1.36  0.94 – 1.28 |
| **Wage**  0-2,000  2,001-4,000  4,001+ |  |  |  |  | Ref  0.80***  0.59*** | 0.71 – 0.90  0.44 – 0.80 |
| **Full-time/part-time employment**  Part-time  Full-time |  |  |  |  | Ref  0.64*** | 0.57 – 0.71 |
| **Overtime**  No overtime  Overtime |  |  |  |  | Ref  1.24*** | 1.13 – 1.37 |
| **Night work**  No  Yes |  |  |  |  | Ref  0.94 | 0.82 – 1.08 |

*p****^1^****: significance p*≤ 0.1 | p**≤ 0.05 | p***≤ 0.01 Wald test*

*Unadjusted: commuting status.*

*Adjusted: commuting status, sex, age, education, occupational category, father’s occupational category, born abroad, cohabiting, children, departments, urban area.*

*Fully adjusted: commuting status, sex, age, education, occupational category, father’s occupational category, born abroad, cohabiting, children, departments, urban area, permanency of the job, sector, number of people employed at the local unit, wage, full-time/part-time employment, overtime, night work.*

| **Activity limitation** | **Unadjusted** |  | **Adjusted** |  | **Fully adjusted** |  |
| --- | --- | --- | --- | --- | --- | --- |
| **Variables** | **OR** | **95%CI** | **OR** | **95%CI** | **OR** | **95%CI** |
| **Commuting status**  NCBW  CBW | Ref  0.73*** | 0.62 – 0.85 | Ref  0.74*** | 0.63 – 0.87 | Ref  0.85* | 0.71 – 1.01 |
| **Sex**  Women  Men |  |  | Ref  0.87*** | 0.79 – 0.96 | Ref  1.03 | 0.92 – 1.15 |
| **Age**  20-29  30-39  40-49  50-60 |  |  | Ref  1.77***  2.71***  4.03*** | 1.46 – 2.14  2.28 – 3.23  3.41 – 4.75 | Ref  1.77***  2.71***  4.04*** | 1.46 – 2.16  2.26 – 3.25  3.39 – 4.81 |
| **Education**  Up to secondary school  Up to Bachelor’s degree  Master’s degree & above |  |  | Ref  0.75***  0.68*** | 0.66 – 0.86  0.55 – 0.85 | Ref  0.75***  0.68*** | 0.65 – 0.85  0.55 – 0.85 |
| **Occupational category**  Blue collars  Employees  Intermediates professions  White collars |  |  | 1.36***  1.20***  Ref  0.73*** | 1.20 – 1.55  1.05 – 1.36  0.61 – 0.88 | 1.37***  1.12*  Ref  0.82** | 1.19 – 1.58  0.98 – 1.28  0.68 – 0.98 |
| **Father’s occupational category**  Not field out  Farmers  Artisans, merchants, company directors  White collars  Intermediates professions  Employees  Blue collars |  |  | 1.25**  0.93  1.10  1.09  Ref  1.09  1.02 | 1.04 – 1.50  0.73 – 1.19  0.91 – 1.34  0.89 – 1.33  0.90 – 1.32  0.89 - 1.18 | 1.25**  0.93  1.12  1.07  Ref  1.10  1.01 | 1.04 – 1.51  0.73 – 1.19  0.92 – 1.36  0.87 – 1.30  0.90 – 1.33  0.88 - 1.16 |
| **Born abroad**  Born in France  Not born in France |  |  | Ref  0.89 | 0.76 – 1.05 | Ref  0.89 | 0.76 – 1.05 |
| **Cohabiting**  Alone  Couple |  |  | Ref  0.90** | 0.80 – 1.00 | Ref  0.90* | 0.81 – 1.00 |
| **Children**  No children  Children |  |  | Ref  0.89** | 0.80 – 0.98 | Ref  0.88** | 0.79 – 0.97 |
| **Department**  Ain 01  Meurthe-et-Moselle 54  Moselle 57  Haut-Rhin 68  Haute-Savoie 74  Territoire de Belfort 90 |  |  | Ref  1.02  1.03  0.98  0.98  0.76 | 0.81 – 1.29  0.83 – 1.28  0.77 – 1.23  0.77 – 1.24  0.50 – 1.16 | Ref  1.01  1.04  0.99  1.01  0.74 | 0.80 – 1.28  0.84 – 1.30  0.78 – 1.25  0.80 – 1.29  0.48 – 1.12 |
| **Urban area**  Rural area  Urban area |  |  | Ref  0.96 | 0.86 – 1.07 | Ref  0.94 | 0.85 – 1.05 |
| **Permanency of the job**  Open ended contract  Fix-term contract  Interim |  |  |  |  | Ref  1.05  0.62*** | 0.87 – 1.26  0.44 – 0.89 |
| **Sector**  Not field out  Agriculture  Industry & construction  Trade, transport, lodging & catering  Information & communication  Finance & insurance  Real estate  Scientific & technical activities  Public administration  Other services |  |  |  |  | 0.96  1.12  0.90  0.83***  0.88  0.96  0.65*  0.87  Ref  0.92 | 0.43 – 2.12  0.69 – 1.82  0.79 – 1.04  0.72 – 0.94  0.55 – 1.41  0.73 – 1.26  0.39 – 1.06  0.72 – 1.04  Ref  0.70 – 1.22 |
| **Number of persons working at the local unit**  Not field out  1-9  10-49  50-499  500+ |  |  |  |  | 1.00  Ref  1.15*  1.38***  1.28*** | 0.79 – 1.26  0.99 – 1.34  1.19 – 1.60  1.07 – 1.52 |
| **Wage**  0-2,000  2,001-4,000  4,001+ |  |  |  |  | Ref  0.84***  0.58*** | 0.74 – 0.95  0.43 – 0.79 |
| **Full-time/part-time employment**  Part-time  Full-time |  |  |  |  | Ref  0.65*** | 0.58 – 0.73 |
| **Overtime**  No overtime  Overtime |  |  |  |  | Ref  1.22*** | 1.09 – 1.35 |
| **Night work**  No  Yes |  |  |  |  | Ref  0.82** | 0.70 – 0.96 |

*p****^1^****: significance p*≤ 0.1 | p**≤ 0.05 | p***≤ 0.01 Wald test*

*Unadjusted: commuting status.*

*Adjusted: commuting status, sex, age, education, occupational category, father’s occupational category, born abroad, cohabiting, children, departments, urban area.*

*Fully adjusted: commuting status, sex, age, education, occupational category, father’s occupational category, born abroad, cohabiting, children, departments, urban area, permanency of the job, sector, number of people employed at the local unit, wage, full-time/part-time employment, overtime, night work.*

| **Chronic diseases** | **Unadjusted** |  | **Adjusted** |  | **Fully adjusted** |  |
| --- | --- | --- | --- | --- | --- | --- |
| **Variables** | **OR** | **95%CI** | **OR** | **95%CI** | **OR** | **95%CI** |
| **Commuting status**  NCBW  CBW | Ref  0.82*** | 0.73 – 0.93 | Ref  0.81*** | 0.71 – 0.91 | Ref  0.87** | 0.75 – 1.00 |
| **Sex**  Women  Men |  |  | Ref  0.90*** | 0.83 – 0.97 | Ref  0.99 | 0.91 – 1.08 |
| **Age**  20-29  30-39  40-49  50-60 |  |  | Ref  1.63***  2.32***  3.41*** | 1.42 – 1.88  2.03 – 2.65  3.00 – 3.88 | Ref  1.65***  2.35***  3.48*** | 1.43 – 1.91  2.05 – 2.71  3.04 – 3.99 |
| **Education**  Up to secondary school  Up to Bachelor’s degree  Master’s degree & above |  |  | Ref  0.84***  0.82** | 0.76 – 0.92  0.69 – 0.98 | Ref  0.83***  0.81** | 0.75 – 0.92  0.68 – 0.96 |
| **Occupational category**  Blue collars  Employees  Intermediates professions  White collars |  |  | 1.07  1.01  Ref  0.86** | 0.96 – 1.19  0.91 – 1.12  0.75 – 0.98 | 1.12*  0.98  Ref  0.91 | 1.00 – 1.26  0.88 – 1.09  0.79 – 1.04 |
| **Father’s occupational category**  Not field out  Farmers  Artisans, merchants, company directors  White collars  Intermediates professions  Employees  Blue collars |  |  | 1.11  0.90  1.02  0.93  Ref  1.03  1.00 | 0.96 – 1.29  0.74 – 1.10  0.88 – 1.20  0.80 – 1.09  0.88 – 1.21  0.90 – 1.12 | 1.12  0.91  1.04  0.92  Ref  1.04  1.00 | 0.96 – 1.30  0.75 – 1.12  0.89 – 1.22  0.79 – 1.08  0.88 – 1.21  0.89 – 1.12 |
| **Born abroad**  Born in France  Not born in France |  |  | Ref  1.02 | 0.89 – 1.15 | Ref  1.02 | 0.90 – 1.16 |
| **Cohabiting**  Alone  Couple |  |  | Ref  0.94 | 0.86 – 1.02 | Ref  0.94 | 0.86 – 1.03 |
| **Children**  No children  Children |  |  | Ref  0.92* | 0.85 – 1.00 | Ref  0.91** | 0.84 – 0.99 |
| **Department**  Ain 01  Meurthe-et-Moselle 54  Moselle 57  Haut-Rhin 68  Haute-Savoie 74  Territoire de Belfort 90 |  |  | Ref  1.15  1.37***  1.18*  1.21*  0.96 | 0.95 – 1.38  1.15 – 1.63  0.98 – 1.42  0.99 – 1.47  0.69 – 1.33 | Ref  1.14  1.39***  1.20*  1.23**  0.95 | 0.95 – 1.38  1.16 – 1.66  0.99 – 1.45  1.01 – 1.50  0.68 – 1.31 |
| **Urban area**  Rural area  Urban area |  |  | Ref  1.04 | 0.96 – 1.14 | Ref  1.03 | 0.94 – 1.12 |
| **Permanency of the job**  Open ended contract  Fix-term contract  Interim |  |  |  |  | Ref  1.09  0.69*** | 0.95 – 1.26  0.52 – 0.92 |
| **Sector**  Not field out  Agriculture  Industry & construction  Trade, transport, lodging & catering  Information & communication  Finance & insurance  Real estate  Scientific & technical activities  Public administration  Other services |  |  |  |  | 0.52*  0.61**  0.84***  0.83***  0.88  0.81*  0.73*  0.83**  Ref  1.04 | 0.26 – 1.05  0.40 – 0.94  0.75 – 0.94  0.75 – 0.93  0.66 – 1.18  0.65 – 1.02  0.52 – 1.03  0.71 – 0.96  0.84 – 1.30 |
| **Number of persons working at the local unit**  Not field out  1-9  10-49  50-499  500+ |  |  |  |  | 1.08  Ref  1.08  1.22***  1.15* | 0.90 – 1.30  0.96 – 1.22  1.08 – 1.37  1.00 – 1.32 |
| **Wage**  0-2,000  2,001-4,000  4,001+ |  |  |  |  | Ref  0.89**  0.84 | 0.81 – 0.98  0.67 – 1.06 |
| **Full-time/part-time employment**  Part-time  Full-time |  |  |  |  | Ref  0.78*** | 0.71 – 0.86 |
| **Overtime**  No overtime  Overtime |  |  |  |  | Ref  1.30*** | 1.19 – 1.41 |
| **Night work**  No  Yes |  |  |  |  | Ref  0.95 | 0.84 – 1.07 |

*p****^1^****: significance p*≤ 0.1 | p**≤ 0.05 | p***≤ 0.01 Wald test*

*Unadjusted: commuting status.*

*Adjusted: commuting status, sex, age, education, occupational category, father’s occupational category, born abroad, cohabiting, children, departments, urban area.*

*Fully adjusted: commuting status, sex, age, education, occupational category, father’s occupational category, born abroad, cohabiting, children, departments, urban area, permanency of the job, sector, number of people employed at the local unit, wage, full-time/part-time employment, overtime, night work.*

| **Disability** | **Unadjusted** |  | **Adjusted** |  | **Fully adjusted** |  |
| --- | --- | --- | --- | --- | --- | --- |
| **Variables** | **OR** | **95%CI** | **OR** | **95%CI** | **OR** | **95%CI** |
| **Commuting status**  NCBW  CBW | Ref  0.40*** | 0.30 – 0.54 | Ref  0.39*** | 0.29 – 0.53 | Ref  0.48*** | 0.34 – 0.67 |
| **Sex**  Women  Men |  |  | Ref  1.09 | 0.93 – 1.28 | Ref  1.58*** | 1.31 – 1.91 |
| **Age**  20-29  30-39  40-49  50-60 |  |  | Ref  2.13***  4.25***  4.18*** | 1.55 – 2.93  3.14 – 5.76  3.12 – 5.60 | Ref  2.19***  4.27***  4.06*** | 1.60 – 2.99  3.16 – 5.78  3.03 – 5.45 |
| **Education**  Up to secondary school  Up to Bachelor’s degree  Master’s degree & above |  |  | Ref  0.63***  0.57*** | 0.51 – 0.78  0.39 – 0.83 | Ref  0.60***  0.54*** | 0.48 – 0.75  0.37 – 0.79 |
| **Occupational category**  Blue collars  Employees  Intermediates professions  White collars |  |  | 1.63***  1.36***  Ref  0.67*** | 1.34 – 1.99  1.11 – 1.67  0.50 – 0.91 | 1.80***  1.16  Ref  0.75* | 1.44 – 2.25  0.94 – 1.43  0.55 – 1.01 |
| **Father’s occupational category**  Not field out  Farmers  Artisans, merchants, company directors  White collars  Intermediates professions  Employees  Blue collars |  |  | 1.21  0.78  0.95  1.01  Ref  0.91  0.99 | 0.91 – 1.60  0.53 – 1.17  0.71 – 1.28  0.73 – 1.38  0.67 – 1.23  0.79 – 1.24 | 1.17  0.77  0.98  0.95  Ref  0.91  0.96 | 0.88 – 1.55  0.52 – 1.15  0.73 – 1.32  0.69 – 1.31  0.67 – 1.24  0.77 – 1.20 |
| **Born abroad**  Born in France  Not born in France |  |  | Ref  0.86 | 0.68 – 1.09 | Ref  0.84 | 0.65 – 1.07 |
| **Cohabiting**  Alone  Couple |  |  | Ref  0.76*** | 0.66 – 0.88 | Ref  0.78*** | 0.67 – 0.91 |
| **Children**  No children  Children |  |  | Ref  0.74*** | 0.64 – 0.86 | Ref  0.74*** | 0.63 – 0.86 |
| **Department**  Ain 01  Meurthe-et-Moselle 54  Moselle 57  Haut-Rhin 68  Haute-Savoie 74  Territoire de Belfort 90 |  |  | Ref  1.46*  1.36*  1.17  1.30  1.17 | 0.99 – 2.18  0.96 – 1.94  0.80 – 1.71  0.88 – 1.93  0.62 – 2.21 | Ref  1.40*  1.33  1.18  1.32  1.11 | 0.94 – 2.08  0.93 – 1.91  0.81 – 1.72  0.89 – 1.96  0.59 – 2.11 |
| **Urban area**  Rural area  Urban area |  |  | Ref  1.06 | 0.90 – 1.26 | Ref  1.02 | 0.86 – 1.21 |
| **Permanency of the job**  Open ended contract  Fix-term contract  Interim |  |  |  |  | Ref  1.16  0.51** | 0.92 – 1.46  0.28 – 0.93 |
| **Sector**  Not field out  Agriculture  Industry & construction  Trade, transport, lodging & catering  Information & communication  Finance & insurance  Real estate  Scientific & technical activities  Public administration  Other services |  |  |  |  | 0.49  0.89  0.64***  0.57***  0.75  1.09  0.83  0.59***  Ref  0.64** | 0.11 – 2.29  0.43 – 1.83  0.52 – 0.80  0.46 – 0.71  0.42 – 1.34  0.71 – 1.68  0.44 – 1.54  0.44 – 0.80  0.42 – 0.97 |
| **Number of persons working at the local unit**  Not field out  1-9  10-49  50-499  500+ |  |  |  |  | 1.66***  Ref  1.24*  1.67***  1.50*** | 1.15 – 2.41  0.96 – 1.60  1.30 – 2.14  1.13 – 2.00 |
| **Wage**  0-2,000  2,001-4,000  4,001+ |  |  |  |  | Ref  0.84  0.52** | 0.68 – 1.04  0.29 – 0.94 |
| **Full-time/part-time employment**  Part-time  Full-time |  |  |  |  | Ref  0.45*** | 0.37 – 0.54 |
| **Overtime**  No overtime  Overtime |  |  |  |  | Ref  1.06 | 0.90 – 1.25 |
| **Night work**  **No Yes** |  |  |  |  | Ref  0.60*** | 0.46 – 0.78 |

*p****^1^****: significance p*≤ 0.1 | p**≤ 0.05 | p***≤ 0.01 Wald test*

*Unadjusted: commuting status.*

*Adjusted: commuting status, sex, age, education, occupational category, father’s occupational category, born abroad, cohabiting, children, departments, urban area.*

*Fully adjusted: commuting status, sex, age, education, occupational category, father’s occupational category, born abroad, cohabiting, children, departments, urban area, permanency of the job, sector, number of people employed at the local unit, wage, full-time/part-time employment, overtime, night work.*

| **No leisure activities** | **Unadjusted** |  | **Adjusted** |  | **Fully adjusted** |  |
| --- | --- | --- | --- | --- | --- | --- |
| **Variables** | **OR** | **95%CI** | **OR** | **95%CI** | **OR** | **95%CI** |
| **Commuting status**  NCBW  CBW | Ref  1.06 | 0.92 – 1.22 | Ref  0.95 | 0.82 – 1.10 | Ref  0.97 | 0.82 – 1.13 |
| **Sex**  Women  Men |  |  | Ref  1.68*** | 1.52 – 1.85 | Ref  1.69*** | 1.51 – 1.89 |
| **Age**  20-29  30-39  40-49  50-60 |  |  | Ref  1.22***  1.34***  1.45*** | 1.05 – 1.42  1.17 – 1.54  1.27 – 1.67 | Ref  1.28***  1.41***  1.55*** | 1.10 – 1.49  1.22 – 1.64  1.34 – 1.80 |
| **Education**  Up to secondary school  Up to Bachelor’s degree  Master’s degree & above |  |  | Ref  0.58***  0.57*** | 0.52 – 0.65  0.49 – 0.67 | Ref  0.60***  0.59*** | 0.54 – 0.68  0.50 – 0.69 |
| **Occupational category**  Blue collars  Employees  Intermediates professions  White collars |  |  | 1.60***  1.27***  Ref  0.89* | 1.38 – 1.86  1.12 – 1.43  0.77 – 1.01 | 1.50***  1.21***  Ref  0.89 | 1.28 – 1.75  1.07 – 1.37  0.77 – 1.03 |
| **Father’s occupational category**  Not field out  Farmers  Artisans, merchants, company directors  White collars  Intermediates professions  Employees  Blue collars |  |  | 1.22**  0.84  0.89  0.82**  Ref  0.97  1.20*** | 1.01 – 1.48  0.65 – 1.08  0.74 – 1.06  0.70 – 0.97  0.80 – 1.17  1.05 - 1.37 | 1.20*  0.83  0.89  0.82**  Ref  0.97  1.19** | 0.99 – 1.45  0.64 – 1.07   - 1. – 1.07   0.70 – 0.96  0.80 – 1.17  1.04 - 1.36 |
| **Born abroad**  Born in France  Not born in France |  |  | Ref  1.40*** | 1.18 – 1.67 | Ref  1.36*** | 1.14 – 1.61 |
| **Cohabiting**  Alone  Couple |  |  | Ref  1.11* | 1.00 – 1.23 | Ref  1.12** | 1.01 – 1.25 |
| **Children**  No children  Children |  |  | Ref  1.04 | 0.94 – 1.14 | Ref  1.04 | 0.94 – 1.15 |
| **Department**  Ain 01  Meurthe-et-Moselle 54  Moselle 57  Haut-Rhin 68  Haute-Savoie 74  Territoire de Belfort 90 |  |  | Ref  1.35***  1.61***  1.25*  0.97  1.62** | 1.07 – 1.69  1.29 – 2.01  0.99 – 1.56  0.78 – 1.22  1.05 – 2.50 | Ref  1.34**  1.58***  1.24*  0.98  1.63** | 1.07 – 1.68  1.26 – 1.97  0.99 – 1.56  0.78 – 1.23  1.06 – 2.51 |
| **Urban area**  Rural area  Urban area |  |  | Ref  0.95 | 0.85 – 1.06 | Ref  0.94 | 0.84 – 1.05 |
| **Permanency of the job**  Open ended contract  Fix-term contract  Interim |  |  |  |  | Ref  1.11  1.00 | 0.94 – 1.31  0.67 – 1.48 |
| **Sector**  Not field out  Agriculture  Industry & construction  Trade, transport, lodging & catering  Information & communication  Finance & insurance  Real estate  Scientific & technical activities  Public administration  Other services |  |  |  |  | 2.66*  1.66*  1.07  1.30***  0.92  1.15  1.13  1.21**  Ref  1.16 | 0.93 – 7.58  0.92 – 3.01  0.93 – 1.24  1.14 – 1.49  0.68 – 1.23  0.92 – 1.44  0.77 – 1.66  1.01 – 1.45  0.93 – 1.45 |
| **Number of persons working at the local unit**  Not field out  1-9  10-49  50-499  500+ |  |  |  |  | 1.81***  Ref  1.11  1.25***  1.27*** | 1.41 – 2.32  0.97 – 1.27  1.09 – 1.43  1.07 – 1.50 |
| **Wage**  0-2,000  2,001-4,000  4,001+ |  |  |  |  | Ref  0.90*  0.91 | 0.80 – 1.02  0.73 – 1.15 |
| **Full-time/part-time employment**  Part-time  Full-time |  |  |  |  | Ref  0.97 | 0.86 – 1.09 |
| **Overtime**  No overtime  Overtime |  |  |  |  | Ref  0.92 | 0.83 – 1.02 |
| **Night work**  No  Yes |  |  |  |  | 0.99 | 0.84 – 1.18 |

*p****^1^****: significance p*≤ 0.1 | p**≤ 0.05 | p***≤ 0.01 Wald test*

*Unadjusted: commuting status.*

*Adjusted: commuting status, sex, age, education, occupational category, father’s occupational category, born abroad, cohabiting, children, departments, urban area.*

*Fully adjusted: commuting status, sex, age, education, occupational category, father’s occupational category, born abroad, cohabiting, children, departments, urban area, permanency of the job, sector, number of people employed at the local unit, wage, full-time/part-time employment, overtime, night work.*

**Table A4: Predicted wages in €, by health index units.**

| **Health index** | **0= low** | **p^1^** | **1** | **p^1^** | **2** | **p^1^** | **3** | **p^1^** | **4** | **p^1^** | **5=high** | **p^1^** | **^Marginal increase^** | | **p^1^** | |  |
| --- | --- | --- | --- | --- | --- | --- | --- | --- | --- | --- | --- | --- | --- | --- | --- | --- | --- |
| NCBWs | 1,369 | *** | 1,569 | *** | 1,800 | *** | 1,808 | *** | 1,826 | *** | 1,957 | *** | 81 | | *** | |  |
| CBWs | 3,358 | *** | 2,954 | *** | 3,059 | *** | 3,349 | *** | 3,375 | *** | 3,790 | *** | 161 | | *** | |  |
| Total | 1,592 | *** | 1,725 | *** | 1,941 | *** | 1,981 | *** | 1,999 | *** | 2,163 | *** | 104 | | *** | |  |
| *p****^1^****: significance p***< 0.01; Student’s t-test* | | | | | | | | | | | | | |  | |  | |

**Table A5: Associations between health limitations and demographic background and labour status variables: summary table**

| **Health outcomes**  **Fully adjusted model** | **Low perceived health** | **Activity limitation** | **Chronic diseases** | **Disability** | **Significance summation** | **Mean coefficient value** |
| --- | --- | --- | --- | --- | --- | --- |
| **Commuting status**  NCBW  CBW | Ref  **-** | Ref  - | Ref  -* | Ref  -* | 2 | 0.77 |
| **Sex**  Women  Men | Ref  + | Ref  + | Ref  - | Ref  +* | 1 |  |
| **Age**  20-29  30-39  40-49  50-60 | Ref  +*  +*  +* | Ref  +*  +*  +* | Ref  +*  +*  +* | Ref  +*  +*  +* | 4  4  4 | 1.86  3.09  4.09 |
| **Education**  Up to secondary school  Up to Bachelor’s degree  Master’s degree & above | Ref  -*  -* | Ref  -*  -* | Ref  -*  -* | Ref  -*  -* | 4  4 | 0.73  0.69 |
| **Occupational category**  Blue collars  Employees  Intermediates professions  White collars | +*  +  Ref  - | +*  +  Ref  -* | +  -  Ref  - | +*  +  Ref  - | 3  0  1 | 1.40 |
| **Father’s occupational category**  Not field out  Farmers  Artisans, merchants, company directors  White collars  Intermediates professions  Employees  Blue collars | +  -  -  -  Ref  -  - | +*  -  +  +  Ref  +  + | +  -  +  -  Ref  +  = | +  -  -  -  Ref  -  - | 1  0  0  0  0  0 |  |
| **Born abroad**  Born in France  Not born in France | Ref  +* | Ref  - | Ref  + | Ref  - | 1 |  |
| **Cohabiting**  Alone  Couple | Ref  -* | Ref  -* | Ref  - | Ref  -* | 3 | 0.87 |
| **Children**  No children  Children | Ref  -* | Ref  -* | Ref  -* | Ref  -* | 4 | 0.86 |
| **Department**  Ain 01  Meurthe-et-Moselle 54  Moselle 57  Haut-Rhin 68  Haute-Savoie 74  Territoire de Belfort 90 | Ref  +  +  +  +  + | Ref  +  +  -  +  - | Ref  +  +*  +  +*  - | Ref  +  +  +  +  + | 0  1  0  1  0 |  |
| **Urban area**  Rural area  Urban area | Ref  + | Ref  - | Ref  + | Ref  + | 0 |  |
| **Permanency of the job**  Open ended contract  Fix-term contract  Interim | Ref  -  - | Ref  +  -* | Ref  +  -* | Ref  +  -* | 0  3 | 0.69 |
| **Sector**  Not field out  Agriculture  Industry & construction  Trade, transport, lodging & catering  Information & communication  Finance & insurance  Real estate  Scientific & technical activities  Public administration  Other services | -  +  +  +  +  -  -  -  Ref  - | -  +  -  +*  -  -  -  -  Ref  - | -  -*  -*  -*  -  -  -  -*  Ref  + | -  -  -*  -*  -  +  -  -*  Ref  -* | 0  1  2  3  0  0  0  2  1 | 0.85  0.81  0.82 |
| **Number of persons working at the local unit**  Not field out  1-9  10-49  50-499  500+ | +  Ref  +*  +*  + | +  Ref  +  +*  +* | +  Ref  +  +*  + | +*  Ref  +  +*  +* | 1  1  4  2 | 1.37  1.26 |
| **Wage**  0-2,000  2,001-4,000  4,001+ | Ref  -*  -* | Ref  -*  -* | Ref  -*  - | Ref  -  -* | 3  3 | 0.84  0.63 |
| **Full-time/part-time employment**  Part-time  Full-time | Ref  -* | Ref  -* | Ref  -* | Ref  -* | 4 | 0.63 |
| **Overtime**  No overtime  Overtime | Ref  +* | Ref  +* | Ref  +* | Ref  - | 3 | 1.20 |
| **Night work**  No  Yes | Ref  - | Ref  -* | Ref  -* | Ref  -* | 3 | 0.83 |

- *CI excludes the value 1*

**Table A6: Associations between commuting status and health outcomes. (Last interrogation)**

| **NBCWs = reference group value 1** | |  |  |  |  |  |  |  |  |  |
| --- | --- | --- | --- | --- | --- | --- | --- | --- | --- | --- |
| **Model of regression** | **DE CBWs** | **p^1^** | **BE CBWs** | **p^1^** | **CH CBWs** | **p^1^** | **LU CBWs** | **p^1^** | **Total CBWs** | **p^1^** |
| **Unadjusted** |  |  |  |  |  |  |  |  |  |  |
| Low perceived health | 0.91 (0.59 - 1.41) | NS | 0.61 (0.40 – 0.94) | ** | 0.65 (0.49 – 0.85) | *** | 0.70 (0.49 – 0.99) | ** | **0.66 (0.55 – 0.79)** | ******* |
| Activity limitation | 0.60 (0.32 – 1.11) | NS | 0.60 (0.35 – 1.02) | * | 0.57 (0.42 – 0.78) | *** | 0.73 (0.49 – 1.08) | NS | **0.63 (0.51 – 0.78)** | ******* |
| Chronic diseases | 1.19 (0.82 – 1.73) | NS | 0.55 (0.37 – 0.83) | *** | 0.74 (0.59 – 0.91) | *** | 0.77 (0.58 – 1.02) | * | **0.78 (0.67 – 0.90)** | ******* |
| Disability | 0.09 (0.01 – 0.63) | ** | 0.57 (0.25 – 1.30) | NS | 0.32 (0.13 – 0.76) | *** | 0.59 (0.33 – 1.05) | * | **0.42 (0.27 – 0.65)** | ******* |
| No leisure activities | 0.83 (0.54 – 1.27) | NS | 1.51 (0.94 – 2.43) | * | 1.00 (0.80 – 1.26) | NS | 1.71 (1.13 – 2.58) | ** | **1.18 (0.99 – 1.39)** | ***** |
| **Adjusted** |  |  |  |  |  |  |  |  |  |  |
| Low perceived health | 0.67 (0.43 – 1.05) | * | 0.58 (0.37 – 0.91) | ** | 0.63 (0.48 – 0.83) | *** | 0.68 (0.47 – 0.98) | ** | **0.66 (0.54 – 0.79)** | ******* |
| Activity limitation | 0.46 (0.25 – 0.85) | ** | 0.59 (0.34 – 1.01) | * | 0.55 (0.40 – 0.75) | *** | 0.74 (0.49 – 1.10) | NS | **0.59 (0.48 – 0.74)** | ******* |
| Chronic diseases | 0.95 (0.65 – 1.39) | NS | 0.57 (0.37 – 0.86) | *** | 0.76 (0.61 – 0.95) | ** | 0.87 (0.65 – 1.17) | NS | **0.78 (0.67 – 0.90)** | ******* |
| Disability | 0.07 (0.01 – 0.50) | *** | 0.50 (0.21 – 1.16) | NS | 0.32 (0.13 – 0.82) | ** | 0.55 (0.29 – 1.02) | * | **0.41 (0.25 – 0.65)** | ******* |
| No leisure activities | 0.62 (0.39 – 0.98) | ** | 1.32 (0.82 – 2.12) | NS | 0.95 (0.75 – 1.20) | NS | 1.40 (0.91 – 2.15) | NS | **1.06 (0.89 – 1.26)** | **NS** |
| **Fully adjusted** |  |  |  |  |  |  |  |  |  |  |
| Low perceived health | 0.72  (0.46 – 1.13) | NS | 0.63  (0.40 – 1.01) | * | 0.76  (0.55 – 1.04) | * | 0.79  (0.54 – 1.15) | NS | **0.76**  **(0.62 – 0.92)** | ******* |
| Activity limitation | 0.54  (0.29 – 1.02) | * | 0.68  (0.39 – 1.17) | NS | 0.54  (0.36 – 0.80) | *** | 1.06  (0.69 – 1.64) | NS | **0.69**  **(0.55 – 0.87)** | ******* |
| Chronic diseases | 0.98  (0.67 –1.44) | NS | 0.59  (0.38 –0.89) | ** | 0.70  (0.53 – 0.92) | *** | 0.98  (0.71 – 1.35) | NS | **0.79**  **(0.67 – 0.93)** | ******* |
| Disability | 0.09  (0.01 – 0.62) | ** | 0.63  (0.27 – 1.48) | NS | 0.33  (0.17 – 0.64) | *** | 0.82  (0.41 – 1.65) | NS | **0.50**  **(0.33 – 0.76)** | ******* |
| No leisure activities | 0.55 (0.34 – 0.87) | ** | 1.36 (0.85 – 2.19) | NS | 1.14 (0.86 – 1.50) | NS | 1.39 (0.89 – 2.19) | NS | **1.16 (0.96 – 1.40)** | **NS** |
| **N** | **175** |  | **219** |  | **1,120** |  | **500** |  | **2,037** |  |
| *p****^1^****: significance p*≤ 0.1 \| p**≤ 0.05 \| p***≤ 0.01; Wald test.*  *Unadjusted: commuting status*  *Adjusted: commuting status, sex, age, education, occupational category, father’s occupational category, born abroad, cohabiting, children, departments, urban area*  *Fully adjusted: commuting status, sex, age, education, occupational category, father’s occupational category, born abroad, cohabiting, children, departments, urban area, permanency of the job, sector, number of people employed at the local unit, wage, full-time/part-time employment, overtime, night work* | | | | | | | | |  |  |
